# The impact of autonomy-supportive organizational environments on employees’ emotions and creative performance: A self-determination theory perspective

**DOI:** 10.1371/journal.pone.0322184

**Published:** 2025-05-29

**Authors:** Lan Ye, Yanwei Li, Na Zhang

**Affiliations:** 1 College of Cabin Crew, Civil Aviation University of China, Tianjin, China; 2 School of Economics and Management, Civil Aviation University of China, Tianjin, China; 3 School of Business, Beijing Information Science and Technology University, Beijing, China; 4 Beijing Knowledge Management Research Center, Beijing, China; National University of Modern Languages, PAKISTAN

## Abstract

The ongoing debate over whether positive or negative emotions foster creative performance remains a pivotal issue in understanding the interplay between emotions and creativity. Emerging research suggests that both positive and certain negative emotions, such as fear and guilt, can enhance creativity under specific conditions. Grounded in Self-Determination Theory (SDT), this study examines how autonomy-supportive organizational environments contribute to the satisfaction of employees’ basic psychological needs. It further explores how these needs influence work-related emotions and ultimately foster creative performance. Data were collected from 283 leaders and employees across various enterprises in Mainland China. Descriptive statistics were analyzed using SPSS 26.0, and structural equation modeling (SEM) with latent variables was conducted using AMOS 26.0 to test the hypothesized relationships and mediating effects. The results demonstrate that autonomy support positively influences the satisfaction of basic psychological needs, which subsequently promotes positive emotions and enhances creative performance. Conversely, autonomy support negatively affects the frustration of basic psychological needs, thereby mitigating negative emotions. Mediation analyses reveal that basic psychological needs mediate the relationship between autonomy support and emotions, while both positive and negative emotions mediate the relationship between autonomy support and creative performance. These findings provide valuable insights into the mechanisms linking autonomy-supportive environments, psychological needs, emotions, and creativity. Beyond its theoretical contributions to SDT, this study offers practical guidance for organizations aiming to cultivate employee creativity and well-being by fostering supportive and autonomy-oriented workplace climates.

## Introduction

In the face of escalating global competition and rapid technological advancements, organizations are encountering unprecedented levels of environmental uncertainty. Within this dynamic context, fostering creativity and innovation among employees has become a paramount objective for human resource management [1,2]. Organizational creativity is a cornerstone of overall performance, with employee creativity serving as a key determinant of organizational creative outcomes [3–5]. According to the componential theory of creativity, the external environment influences creativity through affective and motivational mechanisms [6]. Therefore, understanding these affective mechanisms is essential for comprehending the relationship between the environment and creative performance.

Over the past three decades, organizational behavior research has primarily examined the direct impact of employee emotions on workplace creativity, particularly focusing on the roles of both positive and negative emotions in facilitating creative performance [7,8]. However, research findings in this area have been inconsistent [9,10]. While some studies suggest that positive emotions enhance creativity and negative emotions hinder it [11–13]. Additionally, some researchers propose that positive and negative emotions can coexist, collectively influencing creative performance [14–16]. Different positive and negative emotions may exert varying effects at different stages of the creative process [17], and various sub-emotions provide distinct raw materials, activating different cognitive networks and eliciting different creative responses [18]. Therefore, both positive and negative emotions have the potential to facilitate creativity. Despite significant scholarly attention, understanding the influence of emotions on creativity within the workplace remains limited [19], and no consensus has been reached on this issue to date.

The influence of emotions on employees’ creative performance is contingent upon the organizational environment that elicits these emotional experiences [19]. According to the Mood-as-Input model, emotions provide individuals with information, and the significance and impact of this information depend on the surrounding environment [20]. The environment shapes how emotional information is interpreted and its perceived importance. Only by examining the interaction between emotions and specific organizational contexts can definitive conclusions be drawn regarding whether positive or negative emotions enhance employees’ creative performance [21]. Therefore, investigating the antecedents of the work environment is crucial for developing a model that delineates how work environment factors influence employees’ emotions and creative performance, thereby shedding light on the complex relationship between emotions and employee creativity.

An autonomy-supportive environment is a key outcome of Self-Determination Theory (SDT) [22]. SDT posits that the social environment enhances intrinsic motivation and fosters the internalization of extrinsic motivation by satisfying three fundamental psychological needs: autonomy, competence, and relatedness. This leads to increased trust, job satisfaction, a sense of responsibility, and improved job performance within organizations [23]. Autonomy support involves individuals perceiving that they have the freedom to make choices, receive valuable information, have their emotional experiences acknowledged, and face minimal pressure from significant others (e.g., leaders, teachers, parents) [22]. In the workplace, autonomy-supportive environments include leadership support, colleague support, resource availability, customer support, and more—all of which contribute to fulfilling employees’ needs for autonomy, competence, and relatedness. This fulfillment fosters positive work emotions and enhances creative performance. In an autonomy-supportive environment, the satisfaction of basic psychological needs leads to an overall positive affect [24] and increased vitality [25], which are likely to promote enthusiasm [26]. This enthusiasm, in turn, provides the “energy” necessary for creativity. Moe and Katz demonstrated that need satisfaction is linked to the adoption of autonomy-supportive behavior and enthusiasm [27]. Similarly, Aldrup et al. found that perceived need satisfaction leads to increased enthusiasm, which subsequently enhances creativity [28]. Deci and Ryan propose that autonomy-supportive environments in organizations involve presenting employees’ viewpoints, acknowledging their feelings, providing work-related choices and information, and minimizing pressure and demands [29]. Autonomy support encourages leaders to offer information from an acknowledgment perspective, using non-controlling methods to provide choices, deliver feedback, and inspire self-regulation, rather than imposing specific actions [30]. SDT explains that autonomy-supportive environments cultivate intrinsic motivation by fostering the internal, constructive enhancement and integration of individuals’ consciousness. Behaviors driven by intrinsic motivation are more intense and enduring than those driven by extrinsic motivation. Therefore, under SDT, autonomy-supportive environments are more effective in stimulating employee creativity. As a result, the autonomy-supportive work environment emerges as one of the most critical factors influencing organizational creativity.

In recent years, the impact of organizational environments on employees’ well-being and performance has garnered increasing attention in organizational psychology. Specifically, autonomy-supportive organizational climates have been recognized as crucial factors influencing employees’ emotions and creative performance [31–34]. According to Self-Determination Theory (SDT), autonomy support within the workplace enhances individuals’ intrinsic motivation and satisfaction, fostering an environment conducive to creativity and emotional well-being. Despite extensive research highlighting the global trends in autonomy support and its effects on employee emotions and creative performance, the majority of studies have been conducted within Western contexts, leaving a gap in understanding how these dynamics unfold in non-Western cultures. Research has suggested that cultural, social, and organizational factors may shape the ways in which autonomy-supportive environments impact employees’ emotions and creativity [35]. As a result, it is essential to contextualize these findings within specific local settings. This study aims to address this gap by investigating the effects of autonomy-supportive organizational environments on employees’ emotions and creative performance within the context of China, where unique cultural values and organizational structures may influence the outcomes in distinct ways [33,36]. By focusing on this context, this paper seek to provide insights into the nuanced mechanisms through which autonomy support operates in diverse organizational cultures, thereby contributing to both theoretical advancements in SDT and practical implications for organizational management.

Despite previous research on the relationship between affect and creative performance, three significant research gaps remain unaddressed. First, the unresolved debate regarding the role of emotions in creative performance persists. While positive emotions are generally linked to enhanced creativity, certain negative emotions, such as fear or guilt, have also been shown to foster creativity in specific contexts. However, these findings are inconsistent, and the precise emotional states that promote creativity remain unclear. This study aims to address this gap by examining how both positive and negative emotions, within the context of an autonomy-supportive work environment, influence creative performance. By investigating the mediating role of basic psychological need satisfaction in this relationship, this paper hope to clarify the complex dynamics of emotions and their contribution to creativity in the workplace.

Second, there is a need for further exploration of the theoretical framework and the existing research gaps. Although Self-Determination Theory (SDT) has been widely applied to understand motivation and behavior across various contexts, the intersection of SDT, emotions, and creativity within organizational environments has not been thoroughly examined. Specifically, the mediating role of basic psychological need satisfaction in the relationship between autonomy support and creative performance remains underexplored. This study seeks to fill this gap by exploring how autonomy-supportive work environments influence employees’ basic psychological needs, emotions, and creative outcomes, ultimately fostering enhanced creativity. Through this research, this paper aim to provide a deeper understanding of how autonomy support translates into pathways that enhance creative performance.

Third, the specificities of the Chinese organizational context warrant further consideration. With China’s rapid economic growth, the unique organizational dynamics offer a distinctive setting for examining the relationship between autonomy support, psychological needs, and creative performance. Specifically, Chinese organizational culture tends to be more hierarchical, with traditional values emphasizing collective harmony and obedience to authority. As such, the impact of autonomy support on emotions and creativity may differ in China compared to Western contexts, where individual autonomy is more highly prioritized. Understanding this cultural context is crucial for both theoretical and practical reasons: it not only helps assess the universality of SDT across different cultural backgrounds, but also provides valuable insights into how autonomy support can be leveraged to promote creativity within Chinese organizational environments.

This study seeks to employ Self-Determination Theory (SDT) as a guiding framework, focusing on autonomy-supportive organizational environments to examine how work emotions influence employees’ creative performance. By doing so, the study aims to address existing discrepancies in research on emotions and creativity. Additionally, the adoption of a multi-level research approach will allow for an examination of how organizational-level autonomy-supportive environments influence the relationship between individual-level emotions and creative performance, by acting as a contextual factor that shapes emotional responses and their impact on creativity. This comprehensive approach promises to provide a more holistic and integrated perspective compared to previous research efforts.

This study makes several important contributions to the literature. Firstly, it extends Self-Determination Theory (SDT) into the realm of emotion and creativity, offering new insights into how autonomy-supportive organizational environments influence both positive and negative emotions, and how these emotions subsequently impact creativity. By doing so, the study bridges an important gap in the SDT literature, particularly in the context of Eastern cultural backgrounds. While SDT has been widely applied to understanding motivation and behavior, this study is one of the first to explore the applicability of emotions as a mediating variable in SDT within the unique cultural environment of China, where collectivist values and hierarchical organizational structures may influence the dynamics of emotion and creativity. This exploration broadens the theoretical scope of SDT, providing new perspectives on the relationships between autonomy support, emotional states, and creative performance in specific organizational settings.

Secondly, this study contributes to refining and expanding SDT by validating the role of autonomy-supportive environments in fostering positive emotional experiences and creativity. It constructs a causal model that explains how external environmental factors, such as autonomy support, influence individual creativity through the mediation of basic psychological needs and emotional states. This model enhances the theoretical foundations of SDT by demonstrating how different elements of the work environment can shape emotional and creative outcomes. The study also offers critical insights into the role of emotions—both positive and negative—as pivotal in mediating this relationship, highlighting the nuanced role emotions play in creativity across diverse cultural contexts.

From a practical standpoint, this study provides valuable insights for managers in Chinese enterprises, offering strategies to create organizational environments that foster employee creativity. It emphasizes the importance of aligning organizational practices with employees’ psychological needs, suggesting that by fostering a supportive work environment that nurtures moderate emotional experiences, managers can enhance creativity. The findings not only contribute to SDT’s theoretical development but also offer actionable guidance on how managers can cultivate a creativity-enhancing environment within Chinese organizations, which may have cultural dynamics distinct from those in Western settings.

## Literature review and hypotheses

### Creativity performance and emotions

Creativity refers to an individual’s ability to generate new or original ideas, gain insights, recombine existing knowledge, or invent products recognized by experts as having scientific, aesthetic, social, or technological value [4,37]. Employee creativity performance is typically defined as the generation of novel and valuable ideas, perspectives, and problem-solving approaches [38,39]. Emotions are characterized as the attitudes, experiences, and behavioral patterns individuals exhibit in response to perceived external stimuli and are commonly used to explain individual behavior [40]. Organizations are environments saturated with emotions, which permeate the entirety of the work process for employees. Among the various factors predicting employee creativity, emotions are both widely acknowledged and subject to significant debate [41,42].

The central controversy in this research question revolves around the influence of positive and negative emotions on creativity performance [43]. Some scholars argue that positive emotions can significantly enhance creativity [44,45]. For instance, Isen [46] induced positive affect in college students through methods such as small gifts and comedy clips, observing that these students generated more novel and appropriate word associations, thus confirming a positive correlation between positive affect and individual creativity. Madjar and Oldham found that a positive mood mediated the relationship between supportive work/non-work environments and creativity [47]. Additionally, Davis conducted a meta-analysis of 62 experiments and 10 field studies, revealing that among positive, neutral, and negative moods, a positive mood most significantly fosters creativity [48]. However, the impact of positive emotions also depends on the type of creative task [35,49,50]. In contrast, other scholars contend that negative emotions can also enhance creativity. For example, Ludwig’s research on 1,005 outstanding individuals from various professions in the 20th century found a significant positive correlation between depressive emotions and creative achievement [51]. Furthermore, Benoit and Miller demonstrated that fear can enhance creativity perception [15]. Similarly, W. Liu and Xiang showed that negative emotions such as disappointment and shame can positively impact learning and knowledge growth, which benefits creative performance [16].

The organizational field has yet to reach a consensus on the correlation between emotions and creative performance. This lack of agreement largely arises from the fact that employees’ creative endeavors occur within the context of their daily work routines, which are intricately intertwined with broader life objectives such as personal interests, aspirations, recognition, advancement opportunities, and interpersonal dynamics. These activities are enduring, multifaceted, and influenced by contextual elements. Consequently, research exploring the link between emotions and creative performance can only yield conclusive insights when conducted within the specific context of an organization. Findings from studies conducted in other contexts may only suggest the potential relationship between these variables.

### Autonomy-supportive environment

Self-Determination Theory (SDT) provides a comprehensive framework for understanding the relationship between environmental factors and individual behavior [52]. At the core of SDT is the concept of autonomy support, which refers to the ability of authority figures, such as leaders, to consider others’ viewpoints, provide meaningful information and choices, and foster self-determination [29]. This definition outlines four key requirements for authority figures: first, to offer clear explanations for expected behaviors; second, to empathize with others’ perspectives; third, to promote opportunities for choice and initiative; and finally, to minimize controlling behaviors (Landry R, Joussemet M, Koestner et al. [Unpublished]).

Building on this theoretical foundation, Deci and Ryan classified environments into autonomy-supportive and controlling categories based on their effects on individual behavior [22]. Autonomy-supportive environments encourage individuals’ autonomous actions and provide supportive conditions, whereas controlling environments impose external constraints and coercive elements on individuals’ behavior. These environments exert distinct influences on individuals’ psychological experiences and behaviors. Autonomy-supportive environments facilitate the internalization of both intrinsic and extrinsic motivation, while controlling environments undermine intrinsic motivation and hinder the internalization of extrinsic motivation. According to this theory, the fulfillment of individuals’ needs for competence, relatedness, and autonomy serves as the criteria for distinguishing between autonomy-supportive and controlling environments. The concept of autonomy support not only underscores the importance of autonomy as one of the three basic needs for individual development but also highlights the constructive role of environmental support in this development [53], establishing a close link between individual needs and the environment. SDT posits that individuals are inclined to engage in work and tasks that align with their values and interests, yet their motivation and behavior are also influenced, to some extent, by the social environment [33]. Following the logic of SDT, in a controlling organizational environment, employees’ internal motivation and desires are suppressed, negatively impacting their performance and adaptation. Conversely, in an autonomy-supportive organizational environment, employees’ internal motivation and desires are nurtured, leading to improved performance and individual development.

### Autonomy support and basic psychological needs

Autonomy-supportive environments are integral to the satisfaction of basic psychological needs, a cornerstone of Self-Determination Theory (SDT) [29]. SDT posits that the fulfillment of three basic psychological needs—autonomy, competence, and relatedness—plays a fundamental role in motivating individuals and shaping their behavior. These needs represent the core connection between individuals and their external environment. When the environment supports the satisfaction of these needs, it fosters intrinsic motivation, enhances engagement, and improves performance [29].

The need for autonomy is the desire to feel in control of one’s actions and to have the freedom to make choices [54]. Autonomy-supportive environments, where individuals are encouraged to make decisions independently, are believed to fulfill this need and foster autonomous motivation, which is associated with positive outcomes such as higher job satisfaction and enhanced creativity [29].

The competence need refers to the experience of feeling effective in one’s activities and having opportunities to exercise and express one’s abilities [29]. Autonomy-supportive environments play a crucial role in fulfilling this need by providing optimal challenges and constructive feedback, which help individuals feel competent and achieve success. Research has shown that when employees perceive their work environment as supportive of their competence, they are more likely to engage in creative tasks and demonstrate improved performance [29]. Specifically, the presence of autonomy support enhances the competence need by offering challenges that are appropriately matched to individual abilities and providing feedback that fosters a sense of mastery. A study by Van den Broeck et al. further demonstrated that autonomy-supportive climates are positively related to the satisfaction of both competence and autonomy needs, which, in turn, lead to higher levels of intrinsic motivation and better work performance [55].

Relatedness refers to the need to feel connected to others, to build meaningful relationships, and to experience a sense of belonging [29]. Autonomy-supportive environments contribute to the satisfaction of this need by fostering a sense of respect, trust, and collaboration among individuals. When these relatedness needs are met, individuals are more likely to experience positive emotions and increased motivation, which in turn can enhance their creative performance and overall well-being [29]. By creating opportunities for meaningful interpersonal interactions and promoting mutual respect, autonomy-supportive environments facilitate the fulfillment of relatedness needs. Niemann et al. further demonstrated that organizational environments that support autonomy also promote greater satisfaction of relatedness needs, leading to improved employees’ well-being and enhanced creative performance [56].

Deci and Ryan argue that these three psychological needs are universal and innate, and individuals are motivated to seek environments that satisfy them [29]. A supportive social environment that promotes the satisfaction of these needs can lead to positive outcomes, including increased intrinsic motivation, personal growth, and enhanced work performance. Research by Baard et al. found that managers who create autonomy-supportive environments help employees satisfy their basic psychological needs, leading to higher work engagement, greater happiness, and improved performance compared to controlling environments [57].

### Basic psychological needs and emotions

Basic psychological needs are defined as the extent to which the external environment meets individuals’ fundamental psychological needs [58]. Ryan and Deci emphasized the importance of recognizing both the independent functional value of the three basic psychological needs and their dynamic interplay [33]. The satisfaction of these needs is closely linked to the experience of emotions, and previous research has explored the association between the satisfaction of basic psychological needs and both positive and negative emotions. Ryan et al. suggested that greater satisfaction of autonomous psychological needs is associated with increased positive emotions, vitality, and self-esteem, as well as reduced negative emotions and physiological symptoms [59]. A heightened sense of relatedness can also promote physical health. Myers demonstrated that individuals who feel satisfied in their interpersonal interactions tend to be happier and healthier compared to those who do not [60]. In addition to autonomy and relatedness, the satisfaction of competence needs also plays a crucial role in emotional experiences. According to Eddie et al., competence needs are activated when individuals perceive challenges and are able to successfully overcome them. This success triggers positive emotions, such as pride, satisfaction, and confidence, which further enhance intrinsic motivation [61]. When individuals feel competent, their sense of achievement and mastery grows, leading to a positive emotional state that encourages engagement in more challenging tasks and continued growth. These emotions are particularly relevant in contexts where individuals’ sense of competence is nurtured by an autonomy-supportive environment, as the environment provides feedback and optimal challenges that facilitate the satisfaction of competence needs [62].

Furthermore, when the three basic psychological needs—autonomy, competence, and relatedness—are satisfied, individuals report heightened feelings of psychological well-being, joy, and strong engagement in activities. However, when these needs are unsatisfied or partially met, individuals experience negative emotions such as uncertainty, doubt, anger, and fear, and may also suffer from diminished vitality and physical discomfort [63]. Thus, the interplay between these needs, their satisfaction, and the emotional outcomes highlights the importance of an autonomy-supportive environment in promoting positive emotional experiences and enhancing overall motivation.

While Self-Determination Theory (SDT) provides a comprehensive framework for understanding the relationship between autonomy support, psychological needs, and emotional outcomes, alternative theories offer different insights into the interplay between emotions and creativity. For instance, Affective Events Theory (AET) emphasizes the role of workplace events in triggering emotional responses, which then influence employees’ attitudes and behaviors, including creativity [64]. AET posits that emotions are not only consequences of job events but also key drivers of subsequent work behaviors, thus suggesting that both positive and negative emotions can serve as catalysts for creative thinking, depending on the specific work context.

Additionally, Broaden-and-Build Theory of Positive Emotions suggests that positive emotions expand individuals’ thinking and problem-solving capacities, leading to increased creativity [65]. This theory contrasts with SDT by focusing primarily on positive emotions, arguing that they foster a broadened cognitive state that is conducive to novel ideas and flexible thinking. On the other hand, theories such as The Affect-Cognition-Behavior Model propose that emotions serve as an immediate, adaptive response to environmental stimuli that may affect creativity in complex ways [65]. According to Isen, positive emotions often facilitate cognitive flexibility, while negative emotions may enhance critical thinking and attention to detail, potentially improving the quality of creative performance.

Furthermore, The Cognitive Evaluation Theory (CET), a sub-theory of SDT, emphasizes that external factors, including rewards and feedback, can influence intrinsic motivation and emotional responses, which in turn affect creativity [66]. CET suggests that while autonomy support might generally enhance intrinsic motivation and positive emotions, external constraints or controls might lead to frustration and a decrease in creativity. This contrasts with SDT’s broader view of autonomy support fostering positive emotions and creativity in all contexts, highlighting the importance of considering the role of external factors in shaping emotional responses and creative performance.

In sum, while SDT offers a robust framework, integrating these alternative theories and perspectives provides a more nuanced understanding of the complex relationship between emotions and creativity. These competing models highlight that emotions—both positive and negative—can have diverse effects on creativity, depending on individual, contextual, and environmental factors.

### Autonomy support and creative performance

Existing research has identified several environmental factors that influence employees’ creative performance. These factors include supportive and conducive work environments, which encompass elements such as leadership support [67], collegial collaboration [35], an organizational innovation climate [5], expectation management [68], the establishment of creative objectives [69], and the provision of feedback [70], among others. Among these factors, the autonomy-supportive environment stands out as one of the most extensively explored in the research literature.

Numerous studies have demonstrated that autonomy-supportive environments within organizations foster individuals’ intrinsic motivation, job satisfaction, and, in various contexts, enhance job performance. For instance, teachers’ autonomy support positively predicted metacognition, creative thinking, and self-efficacy, with self-efficacy being positively predicted by metacognition and creative thinking [71]. Research indicates that teams led by autonomy-supportive leaders exhibit high levels of trust in the organization and overall job satisfaction [72]. Additionally, Pajak and Glickman observed that autonomy-supportive management practices cultivate trust and loyalty among employees [73]. In a study conducted by Deci and Ryan [74], annual performance evaluation indicators were collected through a questionnaire survey, and Hierarchical Linear Modeling (HLM) was used to examine the SDT model. The results revealed that employees’ self-determined orientation and leaders’ autonomy support independently predicted employees’ psychological needs for autonomy, competence, and relatedness, which, in turn, influenced employees’ job performance and well-being. Although this study did not specifically focus on creativity, its findings align closely with the patterns observed in performance evaluations.

Deci and Ryan emphasize in SDT the pivotal role of leaders’ direct attention to employees’ need for autonomy in shaping environments that foster creativity and individual factors [74]. Within autonomy-supportive environments, individuals demonstrate enhanced cognitive flexibility and experience more positive moods, leading to heightened creativity [22]. Christina et al. assert that most studies provide evidence supporting the positive association between supportive leadership styles and creativity [75]. For instance, Amabile highlights the significant role of autonomy-supportive work environments in nurturing individual creativity within her theory of creativity components [4]. Oldham and Cummings found that autonomy-supportive supervisory styles have a more beneficial impact on subordinates’ creative performance compared to controlling supervisory styles [76]. Gong et al. revealed that leaders’ empowerment encourages individuals to leverage their knowledge and experiences, thereby enhancing their independent and critical thinking abilities [77]. Zhou demonstrated that a highly autonomous environment fosters the generation of creative ideas [70]. Frese found that the more supervisors encourage employees, the more creative suggestions employees offer to the organization [78]. Ford and Kleiner discovered that employees need autonomy in determining how to allocate their work time and conduct their tasks to explore new ideas and foster creativity [79]. Deci et al. observed that under autonomy support, individuals invest more energy in activities, leading to higher levels of creativity [80]. Through a series of experimental studies, both Oldham and Cummings [76] and Zhou [70] found that a supportive, autonomy-oriented leadership style encourages employees to flexibly and persistently acquire innovative ideas and solutions. Additionally, an autonomy-supportive environment promotes the manifestation of employee creativity [81,82]. Conversely, numerous studies have indicated a negative correlation between supervisors’ controlling behaviors (e.g., close supervision) and employee creativity [83]. Researchers have found that when individuals’ work styles are restricted and controlled, and autonomy is diminished, their creativity declines [35].

### Mediating effect of emotions on the relationship between autonomy support and creative performance

Emotional transmission is an additional pathway through which environmental factors, such as autonomy support, influence employees’ creative performance, alongside task motivation mediation [84]. However, research to date has not fully elucidated the emotional dynamics that convey information about autonomy-supportive environments. Only a few studies have indirectly shed light on the impact of autonomy-supportive environments on emotions and creative performance.

Several studies have examined the moderating role of supervisor supportiveness in the relationship between employees’ emotional states and creative performance. Supervisor supportiveness, as defined by George and Zhou [85], involves providing inspirational feedback, nurturing fairness in interpersonal interactions, and earning trust from employees. In environments with high levels of supervisor supportiveness, when creative employees initially experience elevated positive emotional states, subsequent negative emotions become strongly positively correlated with creativity. Conversely, when supervisor supportiveness is low, there is no significant correlation between negative emotions and creativity, even if initial positive emotional scores are high. These findings highlight the significant impact of autonomy-supportive environments on employee emotions and, consequently, creative performance. Deci et al. found through experimental research that an autonomy-supportive management style is associated with higher job satisfaction and improved emotional states across various aspects of work and the work environment [86]. For instance, managers who present job prospects in a non-controlling manner, offer choices, and encourage self-motivation—rather than exerting pressure—foster greater job satisfaction among employees, enhance trust in company management, and evoke other positive work-related emotions, ultimately improving job performance. Breaugh demonstrated that experiencing autonomy in the workplace can increase employee job involvement and performance [87]. Vallerand et al. showed that when subordinates perceive leaders as autonomy-supportive, they exhibit greater job satisfaction, fewer absences, and improved physical and emotional well-being [88].

### Mediating effect of basic psychological need satisfaction on the relationship between autonomy support and creative performance

Autonomy-supportive environments, which facilitate the satisfaction of individuals’ basic psychological needs (autonomy, competence, and relatedness), have been shown to enhance intrinsic motivation and creative performance. Self-Determination Theory (SDT) posits that these needs are essential for fostering autonomous motivation, which is linked to greater psychological well-being and improved performance outcomes [29,67]. Research indicates that the satisfaction of basic psychological needs mediates the relationship between environmental factors, such as autonomy support, and various outcomes, including creativity [59].

The need for autonomy refers to the experience of volition and choice, which is often fulfilled in environments that promote self-direction and personal agency [55]. When autonomy is supported, individuals tend to feel more intrinsically motivated, leading to higher creative performance [35]. Similarly, competence needs are satisfied when individuals feel effective and capable in their tasks, which is particularly relevant in creative work that involves problem-solving and innovation [29]. The satisfaction of competence needs enhances self-confidence and promotes persistence in creative endeavors, leading to higher creative performance [60].

Furthermore, the relatedness need, which pertains to the desire for meaningful social connections and a sense of belonging, also plays a critical role in fostering creativity. When employees perceive their work environment as supportive and collaborative, they are more likely to experience satisfaction of their relatedness needs, which, in turn, can enhance their creativity [35,69]. Therefore, the fulfillment of these three basic psychological needs is crucial in understanding how autonomy-supportive environments contribute to creative performance.

Several studies have demonstrated that the satisfaction of these psychological needs acts as a mediator between autonomy support and creative performance. For instance, research by Van den Broeck et al. revealed that autonomy-supportive climates were positively related to the satisfaction of both competence and autonomy needs, which, in turn, enhanced intrinsic motivation and work performance [56]. Additionally, Baard et al. emphasized that when employees’ basic psychological needs are satisfied, they experience increased engagement and creativity in the workplace [31]. Given the importance of these needs, it is essential to examine how their satisfaction mediates the relationship between autonomy support and creative performance.

### Aims and hypotheses

The purpose of this study is to investigate the impact of employee emotions on creative performance within an autonomy-supportive organizational environment and to develop a model that elucidates the process through which emotions influence creative performance. This study aims to provide insights into how organizational environments and emotional states can be optimized to enhance employees’ creative performance. The primary hypotheses of this investigation are as follows.

The perspective of Self-Determination Theory (SDT) suggests that the degree of need satisfaction is determined by the interaction between individuals and their social environment, serving as direct or interactive predictors of personality traits, behavior, or psychological states [74]. This implies that the influence of social environmental factors on individual personality traits, behavior, or psychological states may not be direct but could be mediated through the satisfaction of basic psychological needs. Ryan and Deci emphasized that when the social environment fulfills basic psychological needs, individuals are more likely to internalize and integrate various aspects of the social world, including external motivations [89]. Conversely, when the social environment hinders the satisfaction of these needs, the efficiency of internalization is compromised. Therefore, this study infers that the impact of an autonomy-supportive work environment on work emotions is likely mediated through the satisfaction of individual basic psychological needs. In other words, the influence of an autonomy-supportive work environment on work emotions is expected to be mediated by the satisfaction of basic psychological needs. If managers’ autonomy support better meets employees’ needs for autonomy, competence, and relatedness, it will lead to greater job satisfaction, improved performance, enhanced persistence, positive acceptance of organizational changes, and better psychological adaptation. An autonomy-supportive environment facilitates the fulfillment of employees’ psychological needs. Therefore, we hypothesize:

**Hypothesis 1a**: Autonomy support is positively associated with basic psychological needs satisfaction.

**Hypothesis 1b**: Autonomy support is negatively associated with basic psychological needs frustration.

Eddie et al. explored the relationship between the satisfaction of basic psychological needs and specific emotions [61]. They used questionnaires to measure psychological needs (competence and relatedness) and emotions (five negative emotions: anger, sadness, fear, guilt, and shame; and one positive emotion: joy), and analyzed the associations between psychological needs and emotions. The findings revealed that competence and relatedness needs were negatively correlated with anger and sadness, positively correlated with joy, and unrelated to guilt and shame. Additionally, the competence need was negatively correlated with fear, and negative emotions were negatively correlated with the satisfaction of competence and relatedness needs. Previous research indicates that the satisfaction of basic psychological needs elicits positive emotions, while the thwarting of these needs triggers negative emotions. Therefore, we hypothesize:

**Hypothesis 2a** Basic psychological needs satisfaction is positively associated with positive emotions.

**Hypothesis 2b** Basic psychological needs frustration is positively associated with negative emotions.

The generation of both positive and negative work emotions is influenced by a dynamic interaction between organizational factors and individual psychological needs. According to Self-Determination Theory (SDT), autonomy-supportive organizational environments that facilitate the satisfaction of employees’ basic psychological needs—autonomy, competence, and relatedness—are critical to fostering intrinsic motivation and positive emotions. When organizational environments nurture these needs, employees are more likely to experience enhanced feelings of satisfaction, pride, and self-efficacy, which in turn promote positive work behaviors such as creativity, engagement, and overall well-being [31,33]. In these supportive settings, employees perceive themselves as competent and autonomous, which strengthens their emotional investment in tasks and increases the likelihood of experiencing positive emotions such as joy, enthusiasm, and fulfillment [25,57].

On the other hand, in environments that lack autonomy support, employees may face frustration in fulfilling their psychological needs. When individuals perceive their needs for competence, autonomy, and relatedness as unmet or thwarted, they are more likely to experience negative emotions such as stress, anxiety, frustration, and disengagement. These negative emotions can reduce motivation and undermine their creative performance, leading to lower job satisfaction and poorer overall well-being [63,73]. Furthermore, the frustration of these needs often triggers defensive behaviors, such as withdrawal or reduced effort, which can impair productivity and innovation [90].

Recent studies have provided compelling evidence that the satisfaction or frustration of these needs serves as a mediating mechanism through which autonomy support influences emotional outcomes in the workplace. For example, a study by Vansteenkiste et al. demonstrated that when employees’ psychological needs are satisfied through autonomy-supportive leadership, they experience higher levels of positive emotions, such as vitality and enthusiasm, which directly enhance their performance [25]. Conversely, the frustration of these needs leads to increased negative emotions, such as irritability and dissatisfaction, which can hinder both personal well-being and organizational performance [91].

The fulfillment or frustration of basic psychological needs plays a crucial role in shaping emotional responses, which in turn impact creative performance. Therefore, investigating the mediating role of need satisfaction is essential for understanding how organizational autonomy support can influence both positive and negative emotions in employees. Based on this theoretical framework, we hypothesize the following:

**Hypothesis 3a** The satisfaction of basic psychological needs mediates the relationship between autonomy support and positive emotions.

**Hypothesis 3b** The frustration of basic psychological needs mediates the relationship between autonomy support and negative emotions.

Amabile et al. concluded that supportive leadership styles more effectively promote employee creativity compared to controlling leadership styles, which undermine it [84]. Zhou argued that when evaluative feedback on initial tasks is provided in a developmental, informational manner, employees’ creative performance improves. Conversely, when leaders adopt a controlling style, employee creativity decreases [70]. In autonomy-supportive environments facilitated by leaders and the broader work context, employees are exposed to supportive, developmental, and informational cues. These cues contribute to the satisfaction of psychological needs, foster internal motivation, and ultimately stimulate creativity. Hence, we propose the following hypothesis:

**Hypothesis 4** Autonomy support positively correlates with creativity performance.

Baard et al. observed that autonomy-supportive leadership facilitates the fulfillment of employees’ basic psychological needs, leading to increased work engagement, higher levels of happiness, and better performance compared to leaders with controlling styles [57]. Moreover, Deci et al. found that perceived autonomy support, as experienced by employees in both state-owned enterprises in Bulgaria and the United States, satisfies employees’ basic psychological needs, elevates work enthusiasm and self-esteem, and reduces job anxiety [29]. These findings suggest that managers who provide methodological guidance in problem-solving, attend to employees’ psychological needs, and foster positive emotions can enhance work performance. Some research found that negative emotions, compared to positive and neutral ones, can be more conducive to creative performance [14–16]. In line with previous research [51], negative emotions, such as anxiety or stress, may serve as a catalyst for creative thinking in certain environments. Specifically, in autonomy-supportive contexts, negative emotions might mediate the relationship between autonomy support and creative performance, as they can drive deeper emotional engagement and cognitive reflection, ultimately enhancing creativity. Therefore, if the autonomy-supportive environment cultivated by managers better satisfies employees’ intrinsic needs, it will lead to increased job satisfaction, perseverance, improved psychological adaptation, heightened positive emotions, reduced negative emotions, enhanced acceptance of organizational changes, and better performance. An autonomy-supportive environment benefits employees’ emotional states and creative performance. Consequently, we propose the following hypotheses:

**Hypothesis 5a** Positive emotions mediate the relationship between autonomy support and creative performance.

**Hypothesis 5b** Negative emotions mediate the relationship between autonomy support and creative performance.

**Fig 1:**
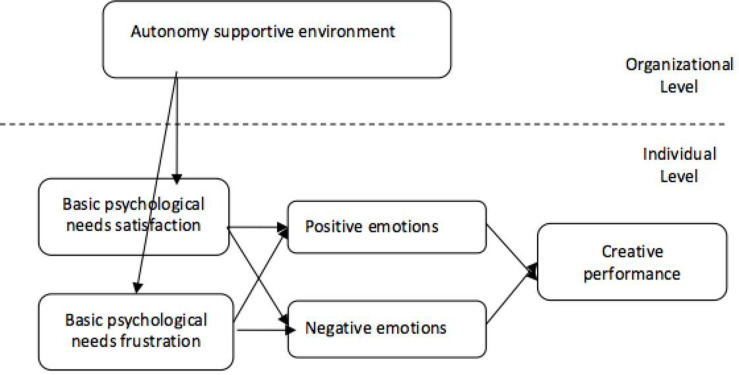
Conceptual model of the study.

The theoretical framework of this study is illustrated in Fig 1. Autonomy support in the workplace—including leadership support, colleague support, resource support, and customer support—facilitates the fulfillment of employees’ needs for autonomy, competence, and relatedness. This, in turn, enhances employees’ positive work emotions and fosters creative performance.

## Method

Before commencing the investigation, this paper obtained ethical approval from the Ethics Committee for Biomedical Research of the Medical College at Hebei University of Engineering (Approval No. 2023[K]030−20). Written informed consent was secured from all participating employees and their respective managers. Additionally, all participants were informed of their right to withdraw from the survey at any stage.

### Participants and procedures

The data collection process followed the principles of random sampling, involving 283 employees. This approach ensured a wide range of perspectives and experiences from different regions. Our sample included individuals from 23 companies across 63 departments, allowing us to represent various organizational structures and industry sectors. By incorporating such a diverse group, we aimed to enhance the generalizability of our findings and address potential concerns regarding the representativeness of the sample. Data collection took place between July and September 2023. In this study, data were collected using paper-based questionnaires. Regarding the study design, it adopts a cross-sectional approach, as data were gathered at a single point in time. Participants completed questionnaires assessing demographics, emotions, basic psychological needs, and autonomy support based on their experiences over the preceding week. Simultaneously, corresponding managers evaluated their subordinates’ creative performance during the same period. All participants provided informed consent to complete the questionnaires and were assured of their right to withdraw at any time if they felt discomfort. Upon completion, participants received small tokens of appreciation.

The sample size for this study was initially estimated using the formula for sample size calculation in proportion-based studies:

Where:

$n\;=\;\frac{Z^{2}\;\times \;P(1-P)}{E^{2}}$

*n* is the required sample size,

*Z* is the Z-value corresponding to a 95% confidence level (Z = 1.96),

*P* is the estimated population proportion, assumed to be 0.5,

*E* is the margin of error, set to 0.05 (5%).

Based on this formula, the required sample size was calculated to be 385 participants. However, due to practical constraints (such as limited access to participants, time restrictions, and non-response rates), the final sample size collected was 283 valid responses. While this is below the calculated sample size, it still provides a sufficient basis for analysis. The data collected is expected to yield reliable and generalizable results within the study’s scope, given the relatively small margin of error and acceptable confidence level. Of the 283 participants, 258 submitted valid responses, resulting in a response rate of 91.2%. Among these, 205 responses were deemed usable, representing 72.4% of the total participants.

Regarding demographic characteristics, 42.4% of the participants were female, and 57.6% were male. In terms of age distribution, 60.5% were under 30 years old, 33.7% were aged 30–40, 4.4% were aged 41–50, and 1.4% were 51 or older. Regarding educational background, 12.7% had an associate degree or lower, 42.0% had a bachelor’s degree, 43.9% had a master’s degree, and 1.4% held a doctoral degree or higher. In terms of employment duration, 78.0% had less than ten years of experience, 16.6% had ten to twenty years, and 5.4% had over twenty years of experience. Table 1 presents detailed data on these sample characteristics.

**Table 1:** Demographic characteristics (n = 205).

Demographics	Classification	Frequency	Percent	Cumulative Percent
Age (years)	<31	124	60.5	60.5
	31-40	69	33.7	94.2
	41-50	9	4.4	98.6
	>51	3	1.4	100.0
Gender	Male	118	57.6	57.6
	Female	87	42.4	100.0
Education	Junior college	26	12.7	12.7
	Bachelor’s degree	86	42.0	54.7
	Master’s degree	90	43.9	98.6
	Doctoral degree	3	1.4	100.0
Job tenure (years)	<10	160	78.0	78.0
	10-20	34	16.6	94.6
	>20	11	5.4	100.0

### Measures

#### Perceived work autonomy support.

This study employed the Work Climate Questionnaire (WCQ) developed by Deci et al. to measure employees’ perceptions of leadership autonomy support in the workplace [92]. The selection of the WCQ was based on their strong relevance to the research objectives and their established reliability and validity in measuring the constructs central to our study. The WCQ scale was chosen for its ability to assess the work context and the autonomy-supportive environment, which directly aligns with our investigation into autonomy support. The WCQ is designed to assess the extent to which employees perceive leadership autonomy support within specific work contexts and is typically administered to employees in organizational settings. The WCQ has been validated and has demonstrated strong validity in the Chinese context [93]. The questionnaire consists of 15 items measured on a 7-point Likert scale, with responses ranging from 1 (strongly disagree) to 7 (strongly agree). The total average score across all items represents the level of perceived autonomy support by employees, with higher average scores indicating a higher level of perceived autonomy support. The reliability of this questionnaire is excellent, as evidenced by a Cronbach’s alpha coefficient of 0.955. The KMO and Bartlett’s test results yielded a value of.923, indicating that the scale demonstrates strong structural validity.

#### Basic psychological needs.

To assess basic psychological need satisfaction, this paper utilized the Basic Psychological Needs Scale-Revised (BPNS-R) [94]. The BPNS-R has been validated in four languages, including Chinese, and has demonstrated strong validity [94]. The scale consists of three subscales corresponding to the three needs identified in Self-Determination Theory (SDT), with each need assessed through eight items that include a balanced combination of both satisfaction and frustration. The reliability of the scale is indicated by the following Cronbach’s alpha coefficients: for competence satisfaction and frustration,.81 and.88, respectively; for autonomy satisfaction and frustration,.71 and.78, respectively; and for relatedness satisfaction and frustration,.77 and.86, respectively, which all demonstrate high reliability. The KMO and Bartlett’s test results yielded a value of.868, indicating that the scale demonstrates strong structural validity.

#### Emotions.

This study utilized a subset of 26 affect items from the expanded Positive and Negative Affect Schedule (PANAS) [95]. The selection of the PANAS scales was based on their strong relevance to the research objectives and their established reliability and validity in measuring the constructs central to our study. The PANAS scale provides a robust measurement of both positive and negative affect, making it particularly suitable for examining the emotional responses of employees in different work settings. Both scales are widely recognized for their psychometric robustness, and their use in prior research ensures their appropriateness for capturing the key variables in our study. The PANAS scales have been validated multiple times and have demonstrated strong validity in the Chinese language [96]. Participants responded to the items on a scale ranging from 1 (never) to 5 (always). The scale includes 6 activated positive affect items (alert, attentive, excited, happy, enthusiastic, interested; α = .81), 6 activated negative affect items (hostile, afraid, guilty, angry, jittery, anxious; α = .87), 7 low arousal positive affect items (grateful, calm, content, relaxed, hopeful, peaceful, at ease; α = .83), and 7 low arousal negative affect items (sluggish, sad, sleepy, lonely, depressed, bored, disappointed; α = .86). The KMO and Bartlett’s test results yielded a value of.874, indicating that the scale demonstrates strong structural validity.

#### Creative performance.

This paper assessed creativity using supervisor ratings from the Creativity Performance Measure (CPM) [97]. The CPM scale has been validated in Chinese and has shown good reliability [98]. Each employee was rated by their supervisor using a 13-item scale. Responses to the 13 items were provided on a scale ranging from 1 (not at all characteristic) to 5 (very characteristic). The internal reliability of the CPM for this study was.93. The KMO and Bartlett’s test results yielded a value of.846, indicating that the scale demonstrates strong structural validity.

## Results

### Descriptive statistics and correlations

As shown in Table 2, the means, standard deviations, and correlation coefficients for the variables are presented. First, this paper examined the relationship between autonomy support and basic psychological needs. Consistent with the tenets of Self-Determination Theory, psychological need satisfaction exhibited a positive correlation with perceived work autonomy support (r = 0.597, p < 0.01), while psychological need frustration showed a negative correlation with perceived work autonomy support (r = −0.420, p < 0.01), thus supporting Hypothesis 1a and Hypothesis 1b.

**Table 2:** Descriptive Statistics and Correlation Coefficient.

Variables	*M*	*SD*	1	2	3	4	5	6
1. Perceived Work Autonomy Support	79.917	14.647	1					
2. Positive emotions	43.848	11.960	.417**	1				
3. Negative emotions	25.834	11.882	−.229**	−.040	1			
4. Psychological needs satisfaction	47.692	5.911	.597**	.461**	−.307**	1		
5. Psychological needs frustration	27.585	7.476	−.420**	−.326**	.489**	−.556**	1	
6. Creative performance	44.839	5.976	.148*	.205**	−.137*	.156*	−.162*	1

Note:

*p < .05,

**p < .01

Second, this paper assessed the relationship between emotions and basic psychological needs. Psychological need satisfaction demonstrated a positive correlation with positive emotions (r = 0.461, p < 0.01) and a negative correlation with negative emotions (r = −0.307, p < 0.01). Conversely, psychological need frustration exhibited a positive correlation with negative emotions (r = 0.489, p < 0.01) and a negative correlation with positive emotions (r = −0.326, p < 0.01), thereby supporting Hypothesis 2a and Hypothesis 2b.

Third, this paper examined the relationship between perceived work autonomy support and creative performance, revealing a positive correlation (r = 0.148, p < 0.05), which supports Hypothesis 4. Additionally, this paper observed positive correlations between creative performance and positive emotions (r = 0.205, p < 0.01), and negative correlations with negative emotions (r = −0.137, p < 0.05). Creative performance also exhibited positive correlations with psychological need satisfaction (r = 0.156, p < 0.05) and negative correlations with psychological need frustration (r = −0.162, p < 0.05). The Pearson correlation coefficients between the variables range from 0.3 to 0.6, representing moderate effect sizes. These statistical findings provide a solid foundation for further causal relationship analysis.

### Mediation analysis

#### Mediation analysis using the stepwise regression method.

To investigate whether basic psychological need satisfaction mediates the relationship between autonomy support and positive emotions, this paper followed the framework proposed by Baron and Kenny, which outlines the steps for testing mediation effects [99]. First, in Step 1, autonomy support was found to be significantly associated with positive emotions (β = 0.417, p < .01). In Step 2, autonomy support significantly predicted basic psychological need satisfaction (β = 0.597, p < .01). Finally, in Step 3, this paper included both autonomy support and basic psychological need satisfaction in the model to assess the mediating effect. In this step, autonomy support continued to predict positive emotions (β = 0.220, p < .01), but there was a significant decrease from β = 0.417, p < .01 to β = 0.220, p < .01. Therefore, this paper concluded that basic psychological need satisfaction partially mediates the relationship between autonomy support and positive emotions. These results are detailed in Table 3, partially supporting Hypothesis 3a.

**Table 3:** The Mediating Effects of Basic Psychological Needs Satisfaction on Perceived Work Autonomy Support and Positive Emotions.

Variables	Step 1 (Positive Emotions)		Step 2 (Basic Psychological Needs Satisfaction)		Step 3 (Positive Emotions)	
β	Sig	β	Sig	β	Sig
Perceived Work Autonomy Support	0.417^**^	<.001	0.597**	<.001	0.220**	0.004**
Basic Psychological Needs Satisfaction					0.330**	<.001
F value	42.614**		112.653**		32.497**	
Sig.	<.001		<.001		<.001	
Adjusted R^2^	0.169		0.354		0.236	

Note:

*p < .05,

**p < .01

While the results provide partial support for Hypothesis 3a, with basic psychological need satisfaction mediating the relationship between autonomy support and positive emotions, we also observed unexpected findings that warrant further discussion. Specifically, while we hypothesized that need satisfaction would fully mediate the relationship, the results indicate a partial mediation effect. This suggests that other factors, potentially unexamined in this study, could be influencing the relationship between autonomy support and positive emotions.

It is possible that individual differences, such as personality traits or prior experiences, may moderate this relationship in ways not captured by our model. Moreover, situational factors within the work environment, such as organizational culture or the nature of tasks, might play a role in how autonomy support translates into emotional outcomes. Future research could explore these additional variables to gain a more comprehensive understanding of the mechanisms at play.

Additionally, the partial mediation may indicate that while basic psychological needs are crucial, they do not fully account for the emotional responses triggered by autonomy support. This raises important questions about the nature of autonomy support and the complex ways in which it influences employee emotions, suggesting that other emotional or cognitive processes might be involved.

Next, this paper investigated whether basic psychological need frustration mediates the relationship between autonomy support and negative emotions in a similar way to positive emotions. In Step 1, autonomy support was found to be significantly associated with negative emotions (β = −0.229, p < .01). In Step 2, autonomy support significantly predicted psychological need frustration (β = −0.420, p < .01). Finally, in Step 3, this paper included both autonomy support and psychological need frustration in the model to assess the mediating effect. In this step, autonomy support no longer predicted negative emotions (β = −0.028, p > .05), with a significant decrease from β = −0.229, p < .01 to β = −0.028, p > .05. Therefore, this paper concluded that psychological need frustration fully mediates the relationship between autonomy support and negative emotions. These results are detailed in Table 4, supporting Hypothesis 3b.

**Table 4:** The Mediating Effects of Basic Psychological Needs Frustration on Perceived Work Autonomy Support and Negative Emotions.

Variables	Step 1 (Negative Emotions)		Step 2 (Basic Psychological Needs Frustration)		Step 3 (Negative Emotions)	
β	Sig	β	Sig	β	Sig
Perceived Work Autonomy Support	−0.229**	0.001**	−0.420**	<.001	−0.028	0.675
Basic Psychological Needs Frustration					0.477**	<.001
F value	11.233**		43.557**		31.889**	
Sig.	<.001		<.001		<.001	
Adjusted R2	0.048		0.173		0.232	

Note:

*p < .05,

**p < .01

However, although the statistical results show that the mediation effect is significant, it is important to consider the practical significance of the findings. The decrease in the path coefficient from β = −0.229 (p < .01) to β = −0.028 (p > .05) suggests that the effect of autonomy support on negative emotions diminishes significantly when psychological need frustration is introduced as a mediator. This indicates that psychological need frustration plays a crucial role in explaining the relationship between autonomy support and negative emotions.

Nevertheless, the small effect size observed in Step 3 (β = −0.028) raises important questions about the practical implications of this finding. While statistically significant, such a small effect size may have limited practical significance in real-world organizational settings. This suggests that although psychological need frustration is a significant mediator, its actual impact on negative emotions may be relatively modest. Future research could explore the contextual factors or other moderating variables that might amplify or attenuate this effect, providing a more nuanced understanding of how autonomy support influences negative emotions in organizational contexts. In sum, while the statistical findings support the full mediation model, further investigation is necessary to assess the practical relevance of these effects, particularly in terms of their real-world applicability and impact.

To examine whether positive emotions mediate the relationship between autonomy support and creative performance, this paper followed Baron and Kenny’s framework [99]. In Step 1, autonomy support was found to be significantly associated with creative performance (β = 0.148, p < .05). In Step 2, autonomy support significantly predicted positive emotions (β = 0.417, p < .01). Finally, in Step 3, both autonomy support and positive emotions were simultaneously included in the model to assess the mediating effect. In this step, autonomy support no longer predicted creative performance (β = 0.076, p > .05), indicating a significant decrease from β = 0.148, p < .05 to β = 0.076, p > .05. Therefore, this paper concluded that positive emotions fully mediate the relationship between autonomy support and creative performance. These results are outlined in Table 5, supporting Hypothesis 5a.

**Table 5:** The Mediating Effects of Positive Emotions on Perceived Work Autonomy Support and Creative Performance.

Variables	Step 1 (Creative Performance)		Step 2 (Positive Emotions)		Step 3 (Creative Performance)	
	β	Sig	β	Sig	β	Sig
Perceived Work Autonomy Support	0.148*	0.034*	0.417**	<.001	0.076	0.317
Positive Emotions					0.173*	0.023*
F value	4.546*		42.614**		4.956**	
Sig.	0.034*		<.001		0.008*	
Adjusted R^2^	0.017		0.169		0.037	

Note:

*p < .05,

**p < .01

However, while the statistical findings support the conclusion of full mediation, the decrease in the path coefficient from β = 0.148 (p < .05) to β = 0.076 (p > .05) warrants further consideration, particularly in relation to the effect size. Although the mediation effect was statistically significant, the relatively small effect size (β = 0.076) in Step 3 raises questions about the practical implications of this finding. This suggests that the mediation effect of positive emotions, while significant, may have limited practical impact in organizational contexts, particularly when the effect size is small.

The relatively small change in the effect size from Step 1 to Step 3 also indicates that while positive emotions play a crucial role in mediating the relationship between autonomy support and creative performance, other factors not included in the model may contribute to creative performance. Future research could explore additional variables, such as personality traits or organizational climate, which could either amplify or attenuate the effect of autonomy support on creative performance through positive emotions.

In terms of practical significance, while the mediation effect is statistically robust, the modest effect size suggests that the real-world implications may be smaller than expected. Organizations may benefit from autonomy-supportive environments, but the impact on creative performance may be less pronounced than suggested by the statistical results alone. Future studies could also investigate the contextual factors that may influence the strength of this effect, such as industry type or the nature of creative tasks, to better understand the practical relevance of autonomy support for enhancing creative performance.

To assess whether negative emotions mediate the relationship between autonomy support and creative performance, this paper followed the same procedure as with positive emotions. In Step 1, autonomy support was found to be significantly associated with creative performance (β = 0.148, p < .05). In Step 2, autonomy support significantly predicted negative emotions (β = −0.229, p < .01). In Step 3, both autonomy support and negative emotions were included in the model to examine the mediating effect. In this step, autonomy support no longer predicted creative performance (β = 0.123, p > .05), indicating a significant decrease from β = 0.148, p < .05 to β = 0.123, p > .05. Thus, this paper concluded that negative emotions fully mediate the relationship between autonomy support and creative performance. These results are detailed in Table 6, supporting Hypothesis 5b.

**Table 6:** The Mediating Effects of Negative Emotions on Perceived Work Autonomy Support and Creative Performance.

Variables	Step 1 (Creative Performance)		Step 2 (Negative Emotions)		Step 3 (Creative Performance)	
	β	Sig	β	Sig	β	Sig
Perceived Work Autonomy Support	0.148*	0.034*	−0.229**	0.001**	0.123	0.085
Negative Emotions					−0.109	0.126
F value	4.546*		11.233**		3.469	
Sig.	0.034*		0.001**		0.033	
Adjusted R^2^	0.017		0.048		0.024	

Note:

*p < .05,

**p < .01

While these results suggest a full mediation effect of negative emotions, it is important to consider the statistical significance of the findings and their practical implications. The significant decrease in the path coefficient from β = 0.148 (p < .05) to β = 0.123 (p > .05) raises questions about the robustness of the mediation effect. While the initial association between autonomy support and creative performance was statistically significant, the effect size decreased substantially in the final model, leading to the non-significant path (β = 0.123, p > .05). This suggests that the impact of negative emotions as a mediator might be weaker than anticipated, which could affect the practical relevance of this mediation model.

The decrease in effect size highlights a potential gap between statistical significance and practical significance. Although the statistical results suggest a full mediation effect, the modest effect size in the final step (β = 0.123) suggests that the actual impact of negative emotions on the relationship between autonomy support and creative performance may be relatively small. This calls for a more nuanced interpretation of the findings, particularly in organizational contexts where the practical implications of mediation effects can be influenced by factors such as organizational culture, task complexity, and individual differences.

#### Mediation analysis using *the* SEM method.

Baron and Kenny’s stepwise regression method provides a foundational approach to testing mediation; however, it has certain limitations, such as its reliance on sequential significance testing and its inability to directly estimate indirect effects. In contrast, structural equation modeling (SEM) offers a more robust alternative by enabling the simultaneous estimation of direct, indirect, and total effects within a single comprehensive model. To further validate the mediation effects described above and to examine the comprehensive framework illustrating how a supportive environment influences creative performance through affective mechanisms, SEM was employed. This method addresses the limitations of stepwise regression while providing a more nuanced understanding of the relationships among the variables.

Given the prior completion of confirmatory factor analysis on latent variables such as positive and negative affect, SEM was selected as the appropriate method for exploring the causal pathways among these variables. The AMOS structural equation modeling software package was used to construct this integrated model, demonstrating the affective mechanisms through which the autonomy-supportive environment impacts creative performance. The Confirmatory Factor Analysis (CFA) was conducted to evaluate the fit of the measurement model. The results revealed an adequate fit, as indicated by the following fit indices: the chi-square to degrees of freedom ratio (χ²/df) was 1.83, which is below the threshold of 5, suggesting a favorable model fit; the Root Mean Square Error of Approximation (RMSEA) was 0.066, below the acceptable threshold of 0.08, indicating a good fit; and the Normed Fit Index (NFI), Non-Normed Fit Index (NNFI), Comparative Fit Index (CFI), Incremental Fit Index (IFI), and Relative Fit Index (RFI) were 0.90, 0.95, 0.95, 0.95, and 0.90, respectively, all of which meet or exceed the threshold of 0.90, indicating satisfactory fit.

**Fig 2:**
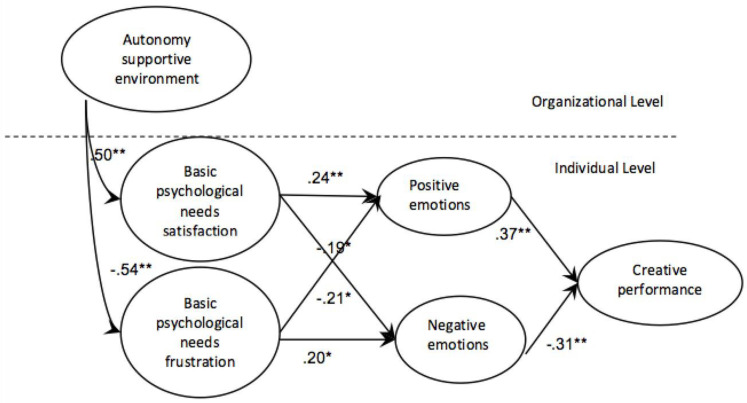
The integrated model of the mechanism.

The structural equation model, based on the proposed framework, is presented in Fig 2. The model was tested to explore the relationships among autonomy support, basic psychological needs satisfaction, frustration, emotions, and creative performance. The results of the structural equation modeling revealed the following relationships: path coefficients were 0.50 (p < 0.01), −0.54 (p < 0.01), 0.24 (p < 0.01), 0.20 (p < 0.05), 0.37 (p < 0.01), and −0.31 (p < 0.01), all of which were statistically significant. These results provide robust support for the proposed model.

To investigate the mediating effects of positive and negative emotions, we tested the indirect relationships between autonomy support and creative performance. These results further support the theoretical framework and the proposed hypotheses 5a and 5b.

## Discussion

In the field of organizational studies, it is well-established that an autonomy-supportive organizational environment positively influences various psychological variables of employees, such as emotions and motivation, as well as outcome variables like creative performance and turnover behavior. This study aims to explore the impact of employee emotions on creative performance within an autonomy-supportive organizational environment and to construct a model that elucidates the mechanisms through which emotions influence creative performance in such an environment. Through this model, the study provides guidance on how organizational context and emotional states can enhance employee creative performance. The main findings of this study are as follows:

The research findings indicate a significant positive correlation between an autonomy-supportive environment and employees’ satisfaction of basic psychological needs, positive emotions, and creative performance. Conversely, there is a significant negative correlation between an autonomy-supportive environment and obstacles to employees’ basic psychological needs, as well as negative emotions. These results align with previous research. Firstly, the findings suggest that an autonomy-supportive environment is positively associated with employees’ positive emotions and negatively associated with their negative emotions. Previous studies have shown that in Western cultural contexts, when managers offer autonomy support through emotional regulation, regular check-ins and feedback, negotiation, and problem-solving, employees exhibit higher performance [37]. According to self-determination theory, an autonomy-supportive environment involves acknowledging employees’ perspectives and providing non-controlling methods, such as relevant information, opportunities for choice, feedback, encouragement for self-initiation, and self-regulation, rather than coercing employees to act in a specific way [30]. This approach fosters positive emotions and mitigates negative emotions among employees, thereby maintaining a relatively healthy emotional state conducive to work.

However, this research also raises questions about the universality of these findings, especially in non-Western cultures. While autonomy-supportive environments are often seen as beneficial in individualistic societies, where personal choice and self-regulation are highly valued, the same mechanisms may not hold in collectivist or hierarchical cultures. In such environments, employees may expect more directive leadership, and an overemphasis on autonomy could potentially lead to role ambiguity, decreased cohesion, or even feelings of insecurity [29]. This contrasts with findings from studies in Eastern cultures, where too much autonomy support has been linked to lower satisfaction and performance due to the cultural preference for more authoritative leadership styles and group-oriented goals [100].

Thus, while the present study’s findings are consistent with previous research in Western contexts, they highlight the need for a more nuanced understanding of the role that cultural values, such as collectivism and power distance, play in shaping the effectiveness of autonomy-supportive environments. Future research should further investigate how these cultural factors moderate the relationship between autonomy support and emotional outcomes, exploring both the positive and potential negative impacts of autonomy in varying cultural settings.

Furthermore, the research results indicate that an autonomy-supportive environment can enhance the satisfaction of employees’ psychological needs. These needs include: Autonomy Needs: The sense of volition and perceived choice in one’s actions, reflecting an individual’s desire for ownership over their behavior. Competence Needs: Feeling effective in one’s actions, being capable of meeting environmental challenges, and achieving desired outcomes. Relatedness Needs: Feeling connected to others and having a sense of belonging within one’s community [29,59]. Deci et al., in their study of employees from state-owned enterprises in Bulgaria and the United States, found that perceived autonomy support in both countries satisfied employees’ basic psychological needs, enhanced their job enthusiasm and self-esteem, and reduced work anxiety [23]. Moreover, the satisfaction of basic psychological needs plays a moderating role in the relationship between autonomy support, motivation, and happiness. When the social environment allows individuals to develop their abilities, connect with others, and act in ways consistent with their self-endorsement, they are more likely to integrate the structures conveyed by the social world into their self-concept. Thus, an autonomy-supportive environment can better fulfill employees’ needs for autonomy, competence, and relatedness, leading to greater job satisfaction, improved job performance, and enhanced psychological adaptation [101].

Thirdly, the research findings indicate that an autonomy-supportive environment can significantly predict employees’ creative performance, which is consistent with previous studies. For example, a study published in 2009 found that autonomy-supportive leadership significantly enhances employees’ intrinsic motivation, which in turn boosts their creative output [102]. Similarly, a meta-analysis of factors influencing creative performance revealed that employees in autonomy-supportive settings tend to exhibit higher levels of creative self-efficacy and innovation [103]. George and Zhou emphasized the importance of autonomy-supportive contexts in the relationship between emotions and creativity [85]. They found that in a supervisor-supportive environment, employees’ positive emotions facilitate divergent thinking, particularly in the early stages of the creative process. Additionally, Martin et al. discovered through a series of experiments that when leaders personally engage with subordinates to discuss and resolve work-related problems, subordinates demonstrate enhanced creative performance [20]. Creativity is more likely to flourish when leaders encourage employees and take steps to help them understand the context and nuances of work-related issues. Leaders who are perceived as autonomy-supportive tend to elicit greater job satisfaction, reduce turnover, and improve the physical and psychological well-being of their subordinates [104]. Therefore, autonomy-supportive leadership and work environments play a crucial role in fostering employees’ creative behaviors.

Fourthly, the research results confirm the mediating role of basic psychological needs in the relationship between an autonomy-supportive environment and work-related emotions. The findings indicate that the satisfaction of basic psychological needs partially mediates the relationship between an autonomy-supportive environment and positive emotions, while the frustration of these needs fully mediates the relationship between an autonomy-supportive environment and negative emotions. These results suggest that an autonomy-supportive environment impacts employees’ levels of psychological need satisfaction and frustration, which in turn generate positive and negative emotions, ultimately increasing the likelihood of creative performance. This highlights the significant causal pathways between employees’ psychological need satisfaction, positive and negative emotions, and creative performance within an autonomy-supportive organizational environment [101,105,106].

Fifthly, the research results confirm the mediating role of work-related emotions in the relationship between an autonomy-supportive environment and creative performance. The findings indicate that both positive and negative emotions fully mediate the relationship between an autonomy-supportive environment and employees’ creative performance. These results suggest that an autonomy-supportive organizational environment influences creative performance through its impact on employees’ emotions, aligning with prior research. Creativity requires intrinsic and sustained motivation to persist through the inherent challenges of creative work, and recent research increasingly focuses on creativity performance within emotional motivation models. Driven by intrinsic motivation, individuals naturally return to the “intuitive domain” of the mind [83]. This unconscious re-entry, characterized by playful thoughts, is crucial for creativity. Amabile et al. found, through the use of the Work Preference Inventory, that participants with a strong orientation toward intrinsic motivation (emotion) exhibit greater creativity [84]. This perspective, rooted in emotional motivation, illustrates how specific organizational contextual factors influence individuals’ intrinsic motivation, ultimately affecting their creative performance. This result not only clarifies the pathway through which an autonomy-supportive environment affects creative performance but also highlights the crucial role of emotions in shaping employee performance within organizational environments.

The findings of this study indicate that emotional states, including both positive and negative emotions, may vary significantly across different age groups and career stages. A notable portion of our sample (60.5%) was under the age of 30, which may have implications for understanding the emotional trajectories of younger individuals in early career stages. Research suggests that individuals in the early stages of their careers are often confronted with different stressors and opportunities compared to those in later career stages. Younger workers may experience a greater sense of novelty and challenges in their work, which could contribute to heightened emotional responses, both positive and negative, as they navigate the demands of professional growth and identity formation [107]. This contrasts with individuals in later career stages who may experience more stability and mastery, potentially leading to lower levels of emotional fluctuation.

Furthermore, the influence of gender on emotional experiences cannot be overlooked. Previous studies have indicated that men and women may experience and express emotions differently due to both biological and social factors [108]. For instance, women are often found to report higher levels of emotional sensitivity and may be more likely to experience intense positive and negative emotions in response to workplace conditions [109]. On the other hand, men may exhibit a different emotional trajectory, influenced by societal norms that encourage emotional restraint. The emotional responses observed in this study could, therefore, reflect the intersection of gender norms, career stage, and the unique challenges faced by individuals at different points in their professional journeys.

These age, career stage, and gender-related variations in emotional experiences may have significant implications for understanding the role of emotions in the workplace. Future research should consider exploring these dimensions further, examining how emotional responses vary not only across career stages but also with respect to the dynamic interactions between age, gender, and work-related factors.

Cultural factors play a crucial role in shaping the dynamics of autonomy support and its effects on emotional outcomes. In the context of this study, we recognize that cultural values, such as collectivism and power distance, significantly influence the relationship between autonomy support and emotions. According to Hofstede’s dimensions of culture [100], Chinese culture emphasizes collectivism, where group goals and harmony often take precedence over individual autonomy. This cultural orientation may moderate how employees perceive and react to autonomy support in the workplace. In such a context, autonomy support might be perceived differently than in individualistic cultures, potentially influencing emotional responses and creative performance. Furthermore, the power distance dimension in Chinese culture, which reflects the extent to which less powerful members of organizations accept unequal power distribution [100], may affect the perceived legitimacy of autonomy support. In high power distance environments, employees may be more reluctant to fully embrace autonomy, as it may conflict with hierarchical expectations and authority. Therefore, while autonomy support is typically associated with positive outcomes such as enhanced emotional well-being and creativity in Western contexts [29], its effects in Chinese organizations may be tempered by these cultural values. This nuanced understanding is crucial for interpreting the generalizability of our findings and contributes to a more culturally informed perspective on organizational behavior.

### Practical implications

This research highlights the significant role of autonomy-supportive environments in enhancing employees’ positive emotions and creative performance. For leaders, this suggests several practical strategies. First, they can foster such environments by offering flexible working hours, enabling remote work options, and cultivating a culture of open discussion and brainstorming [110]. During the initial stages of problem-solving, it is crucial for leaders to refrain from intervening, allowing employees the freedom to express their creative ideas. As the process progresses, leaders should provide the necessary resources and emotional support to ensure that these ideas are effectively implemented. This approach is particularly advantageous for innovative companies that depend on active participation in critical thinking and collaborative brainstorming [111]. For example, providing comfortable workspaces and opportunities for informal interactions can help create an environment conducive to idea exchange and intellectual collaboration.

Furthermore, it is essential to understand that while stressful work environments can also lead to creative performance, they tend to be more suitable for tasks that operate within defined frameworks. In contrast, environments that support autonomy and creativity are better for tasks requiring innovative thinking and problem-solving.

Leaders should also focus on satisfying employees’ intrinsic psychological needs by enhancing their autonomy support. Previous studies indicate that autonomy-supportive behaviors by leaders fulfill employees’ psychological needs, while controlling behaviors diminish motivation and satisfaction [101]. Therefore, effective management should recognize and respect employees’ thoughts and feelings, encourage decision-making, and promote self-initiative to boost creative performance. Tailoring incentive measures to different emotional profiles is key, as positive emotions in autonomy-supportive environments are strongly linked to enhanced creative performance. On the other hand, controlling and pressure-driven management approaches should be avoided, as they may lead to negative emotions that hinder creativity.

To enhance creativity and overall performance within teams, it is essential for managers to foster autonomy-supportive environments and prioritize employees’ emotional well-being. It is crucial to create an emotionally supportive work environment where employees feel comfortable expressing their emotions. Managers can achieve this by being attentive to employees’ emotional states and providing the necessary support, whether through open communication channels, offering emotional resources, or providing stress-relief activities. Supporting employees’ emotional health reduces frustration and promotes overall well-being, which, in turn, enhances their performance and creativity. Autonomy and emotional well-being are closely linked to trust within teams. By cultivating a culture of trust and mutual respect, managers can encourage employees to take creative risks and collaborate effectively. Teams that trust one another are more likely to share innovative ideas and provide constructive feedback, creating a cycle of positive reinforcement for creative thinking. Providing regular feedback and recognition for creative efforts reinforces autonomy and boosts employees’ emotional satisfaction. When employees know their ideas and contributions are valued, they feel more motivated to continue engaging in creative problem-solving. By implementing these strategies, managers can create a work environment that nurtures both autonomy and emotional well-being, ultimately driving higher levels of creativity and improved team performance.

In sum, by creating an autonomy-supportive work environment, leaders can facilitate employees’ emotional well-being and foster higher levels of creativity. This study provides actionable insights that can help organizations improve their management practices and achieve better creative outcomes.

### Limitations

While this study provides valuable insights into employee emotions and their relationship with perceived autonomy support, several limitations should be addressed in future research. Firstly, incorporating additional measures, such as enthusiasm, could offer a more comprehensive understanding of employee motivation dynamics.

Secondly, this study utilized a cross-sectional design, which limits the ability to infer causality or capture changes over time. Future research could employ longitudinal designs to examine how enthusiasm and autonomy support evolve, providing a deeper perspective on their interplay.

Thirdly, while this study focused on employee-reported autonomy support, future investigations could include the perspectives of principals or supervisors, offering a more nuanced understanding of the organizational dynamics influencing employee motivation and autonomy. Addressing these avenues would enhance the robustness and applicability of findings across diverse organizational contexts.

Fourthly, while autonomy-supportive environments are generally beneficial for fostering intrinsic motivation and enhancing employee performance, it is important to recognize their potential drawbacks when over-relied upon. Specifically, an excessive focus on autonomy may lead to role ambiguity or a lack of clear direction within organizations, as employees may struggle to balance their personal autonomy with organizational expectations. In such environments, the absence of sufficient guidance or structure can undermine organizational cohesion, potentially affecting teamwork and collective goals. Moreover, the excessive freedom provided in highly autonomous settings could negatively impact employees’ sense of security or stability, as they may feel disconnected from the broader organizational objectives or experience uncertainty in their roles. This balance between autonomy and structure is critical for maintaining both individual motivation and organizational alignment. We believe this discussion adds depth to our analysis and presents a more nuanced perspective on the limitations of autonomy-supportive environments.

## Conclusion

This study aimed to address the ongoing debate over whether positive or negative emotions promote creative performance. Grounded in Self-Determination Theory (SDT), it examined how an autonomy-supportive organizational environment influences employees’ basic psychological needs, thereby stimulating positive work emotions and ultimately enhancing creative performance. Through a questionnaire survey of corporate employees, the results revealed that an autonomy-supportive environment significantly impacts employees’ satisfaction of psychological needs, as well as their positive and negative emotions and creative performance.

The findings indicate that the satisfaction of employees’ intrinsic psychological needs has a significant influence on both positive and negative emotions, with a particularly strong predictive role in positive emotions. Positive emotions significantly facilitate creative performance, while negative emotions significantly hinder it. The study confirmed that the satisfaction of basic psychological needs partially mediates the relationship between an autonomy-supportive environment and positive emotions, whereas the obstruction of basic psychological needs fully mediates the relationship between an autonomy-supportive environment and negative emotions. Additionally, both positive and negative emotions serve as complete mediating variables between an autonomy-supportive environment and employees’ creative performance.

These results have several practical implications for managers and HR practitioners. Organizations aiming to enhance employee creativity and well-being should prioritize creating autonomy-supportive environments. This includes offering opportunities for choice, providing constructive feedback, and fostering a culture of respect and empowerment. By addressing employees’ psychological needs, managers can enhance positive emotions while mitigating negative emotions, leading to improved creative outcomes.

Future research could explore the nuanced role of cultural and contextual factors in shaping the dynamics between autonomy support, emotions, and creativity. For instance, the impact of autonomy-supportive practices may differ across collectivist versus individualist cultures. Additionally, researchers could investigate how specific negative emotions, such as fear or guilt, might foster creativity under certain circumstances, leveraging findings from recent studies [15,16]. By broadening the scope to include diverse organizational contexts and emotional dynamics, future studies could offer a more comprehensive understanding of how to optimize workplace environments for creativity and innovation.

## References

[1] Lu VN, Wirtz J, Kunz WH, Paluch S, Gruber T, Martins A, et al. Service robots, customers and service employees: what can we learn from the academic literature and where are the gaps?. JSTP. 2020;30(3):361–91. doi: 10.1108/jstp-04-2019-0088

[2] Drazin R, Glynn MA, Kazanjian RK. Multilevel Theorizing about Creativity in Organizations: A Sensemaking Perspective. The Academy of Management Review. 1999;24(2):286. doi: 10.2307/259083

[3] Nasifoglu Elidemir S, Ozturen A, Bayighomog SW. Innovative Behaviors, Employee Creativity, and Sustainable Competitive Advantage: A Moderated Mediation. Sustainability. 2020;12(8):3295. doi: 10.3390/su12083295

[4] Amabile TM, Amabile TM, Collins MA, Conti R, Phillips E, Picariello M, et al. Creativity in Context. Routledge. 2018. doi: 10.4324/9780429501234

[5] Amabile TM. A model of creativity and innovation in organizations. Res Organ Behav. 10:123–67.

[6] Amabile TM, Mueller JS. Studying Creativity, Its Processes, and Its Antecedents. Handbook of Organizational Creativity. Psychology Press. 2024;33–64. doi: 10.4324/9781003573326-3

[7] Zhou J, Shalley CE. Expanding the Scope and Impact of Organizational Creativity Research. Handbook of Organizational Creativity. Psychology Press. 2024;347–68. doi: 10.4324/9781003573326-19

[8] Zenasni F, Lubart TI. Emotion-Related Traits Moderate the Impact of Emotional State on Creative Performances. Journal of Individual Differences. 2008;29(3):157–67. doi: 10.1027/1614-0001.29.3.157

[9] Chattopadhyay P, George E, Li J, Gupta V. Geographical Dissimilarity and Team Member Influence: Do Emotions Experienced in the Initial Team Meeting Matter?. AMJ. 2020;63(6):1807–39. doi: 10.5465/amj.2017.0744

[10] Conroy SA, Becker WJ, Menges JI. The Meaning of My Feelings Depends on Who I Am: Work-Related Identifications Shape Emotion Effects in Organizations. AMJ. 2017;60(3):1071–93. doi: 10.5465/amj.2014.1040

[11] Amabile TM, Pratt MG. The dynamic componential model of creativity and innovation in organizations: Making progress, making meaning. Research in Organizational Behavior. 2016;36:157–83. doi: 10.1016/j.riob.2016.10.001

[12] Xiao F, Wang L, Chen Y, Zheng Z, Chen W. Dispositional and Situational Autonomy as Moderators of Mood and Creativity. Creativity Research Journal. 2015;27(1):76–86. doi: 10.1080/10400419.2015.992683

[13] Shin SJ, Zhou J. Transformational leadership, conservation, and creativity: Evidence from Korea. Academy of Management Journal, 2003;46(6):703–714. doi: 10.5465/30040662

[14] Pessoa L, Engelmann JB. Embedding reward signals into perception and cognition. Front Neurosci. 2010;4:17. doi: 10.3389/fnins.2010.00017 20859524 PMC2940450

[15] Benoit ID, Miller EG. Enhancing creativity perception through fear. Journal of Business Research. 2022;139:1084–98. doi: 10.1016/j.jbusres.2021.10.051

[16] Liu W, Xiang S. The positive impact of guilt. LODJ. 2018;39(7):883–98. doi: 10.1108/lodj-10-2017-0296

[17] SuzanneK.Vosburg GK. “Paradoxical” Mood Effects on Creative Problem-solving. Cognition & Emotion. 1997;11(2):151–70. doi: 10.1080/026999397379971

[18] Lubart TI, Getz I. Emotion, Metaphor, and the Creative Process. Creativity Research Journal. 1997;10(4):285–301. doi: 10.1207/s15326934crj1004_1

[19] Liu W, Li JW, Zhou QW. Cognitive and social mechanisms: the role of emotions in creativity through work-based learning from a functionalist perspective. CMS. 2021;16(2):334–55. doi: 10.1108/cms-02-2020-0049

[20] Martin LL, Ward DW, Achee JW, Wyer RS. Mood as input: People have to interpret the motivational implications of their moods. Journal of Personality and Social Psychology. 1993;64(3):317–26. doi: 10.1037/0022-3514.64.3.317

[21] Martin L, Stoner P. Mood as input: what we think about how we feel determines how we think. Striving and feeling: interactions among goals, affect, and self-regulation. 1996;279–301.

[22] Deci EL, Ryan RM. The support of autonomy and the control of behavior. J Pers Soc Psychol. 1987;53(6):1024–37. doi: 10.1037//0022-3514.53.6.1024 3320334

[23] Deci EL, Ryan RM, Gagné M, Leone DR, Usunov J, Kornazheva BP. Need Satisfaction, Motivation, and Well-Being in the Work Organizations of a Former Eastern Bloc Country: A Cross-Cultural Study of Self-Determination. Pers Soc Psychol Bull. 2001;27(8):930–42. doi: 10.1177/0146167201278002

[24] Stanley PJ, Schutte NS, Phillips WJ. A meta-analytic investigation of the relationship between basic psychological need satisfaction and affect. 2717-7564. 2021;5(1):1–16. doi: 10.47602/jpsp.v5i1.210

[25] Vansteenkiste M, Ryan RM, Soenens B. Basic psychological need theory: Advancements, critical themes, and future directions. Motiv Emot. 2020;44(1):1–31. doi: 10.1007/s11031-019-09818-1

[26] Burić I, Moè A. What makes teachers enthusiastic: The interplay of positive affect, self-efficacy and job satisfaction. Teaching and Teacher Education. 2020;89:103008. doi: 10.1016/j.tate.2019.103008

[27] Moè A, Katz I. Need satisfied teachers adopt a motivating style: The mediation of teacher enthusiasm. Learning and Individual Differences. 2022;99:102203. doi: 10.1016/j.lindif.2022.102203

[28] Aldrup K, Klusmann U, Lüdtke O. Does basic need satisfaction mediate the link between stress exposure and well-being? A diary study among beginning teachers. Learning and Instruction. 2017;50:21–30. doi: 10.1016/j.learninstruc.2016.11.005

[29] Deci E. L., Ryan R. M. The“ what” and” why” of goal pursuits: Human needs and the self-determination of behavior. Psychological inquiry. 2000;11(4):227-–268. doi: 10.1207/s15327965pli1104_02

[30] Deci EL, Spiegel NH, Ryan RM, Koestner R, Kauffman M. Effects of performance standards on teaching styles: Behavior of controlling teachers. Journal of Educational Psychology. 1982;74(6):852–9. doi: 10.1037/0022-0663.74.6.852

[31] Baard PP, Deci EL, Ryan RM. Intrinsic Need Satisfaction: A Motivational Basis of Performance and Weil‐Being in Two Work Settings1. J Applied Social Pyschol. 2004;34(10):2045–68. doi: 10.1111/j.1559-1816.2004.tb02690.x

[32] Gagné M, Deci EL. Self‐determination theory and work motivation. J Organ Behavior. 2005;26(4):331–62. doi: 10.1002/job.322

[33] Ryan RM, Deci EL. Self-determination theory and the facilitation of intrinsic motivation, social development, and well-being. Am Psychol. 2000;55(1):68–78. doi: 10.1037//0003-066x.55.1.68 11392867

[34] Amabile T, Khaire M. Creativity and the role of the leader. Harvard Bus Rev. 2008. 18822674

[35] Zhou J, George JM. When job dissatisfaction leads to creativity: encouraging the expression of voice. Academy of Management Journal. 2001;44(4):682–96. doi: 10.2307/3069410

[36] Hon AHY, Leung ASM. Employee Creativity and Motivation in the Chinese Context: The Moderating Role of Organizational Culture. Cornell Hospitality Quarterly. 2011;52(2):125–34. doi: 10.1177/1938965511403921

[37] Madrid HP, Patterson MG. Creativity at work as a joint function between openness to experience, need for cognition and organizational fairness. Learning and Individual Differences. 2016;51:409–16. doi: 10.1016/j.lindif.2015.07.010

[38] Paulus P, Nijstad B. Group creativity: innovation through collaboration. Oxford University Press. 2003.

[39] Sternberg RJ, Lubart TI. The Concept of Creativity: Prospects and Paradigms. Handbook of Creativity. Cambridge University Press. 1998;3–15. doi: 10.1017/cbo9780511807916.003

[40] Izard CE. Emotion theory and research: highlights, unanswered questions, and emerging issues. Annu Rev Psychol. 2009;60:1–25. doi: 10.1146/annurev.psych.60.110707.163539 18729725 PMC2723854

[41] Ivcevic Z, Moeller J, Menges J, Brackett M. Supervisor Emotionally Intelligent Behavior and Employee Creativity. Journal of Creative Behavior. 2020;55(1):79–91. doi: 10.1002/jocb.436

[42] Liu J, Wang Y, Zhu Y. Climate for innovation and employee creativity. IJM. 2020;41(4):341–56. doi: 10.1108/ijm-02-2017-0030

[43] Grawitch MJ, Munz DC, Kramer TJ. Effects of member mood states on creative performance in temporary workgroups. Group Dynamics: Theory, Research, and Practice. 2003;7(1):41–54. doi: 10.1037/1089-2699.7.1.41

[44] Madrid HP, Patterson MG, Birdi KS, Leiva PI, Kausel EE. The role of weekly high‐activated positive mood, context, and personality in innovative work behavior: A multilevel and interactional model. J Organ Behavior. 2013;35(2):234–56. doi: 10.1002/job.1867

[45] Binnewies C, Wörnlein SC. What makes a creative day? A diary study on the interplay between affect, job stressors, and job control. J Organ Behavior. 2010;32(4):589–607. doi: 10.1002/job.731

[46] Isen AM. On the relationship between affect and creative problem solving. Affect, Creative Experience, and Psychological Adjustment. 1999;3:3–18.

[47] Madjar N, Oldham GR. Preliminary Tasks and Creative Performance on a Subsequent Task: Effects of Time on Preliminary Tasks and Amount of Information About the Subsequent Task. Creativity Research Journal. 2002;14(2):239–51. doi: 10.1207/s15326934crj1402_10

[48] Davis BG. Tools for teaching. John Wiley & Sons. 2009.

[49] Fredrickson BL, Branigan C. Positive emotions broaden the scope of attention and thought-action repertoires. Cogn Emot. 2005;19(3):313–32. doi: 10.1080/02699930441000238 21852891 PMC3156609

[50] Baas M, De Dreu CK, Nijstad BA. A meta-analysis of 25 years of mood-creativity research: Hedonic tone, activation, or regulatory focus?. Psychological bulletin. 2008;134(6):779. doi: 10.1037/a0012815.supp 18954157

[51] Ludwig AM. Creative achievement and psychopathology: comparison among professions. Am J Psychother. 1992;46(3):330–56. doi: 10.1176/appi.psychotherapy.1992.46.3.330 1530096

[52] Deci EL, Ryan RM. Intrinsic Motivation and Self-Determination in Human Behavior. New York: Plenum. 1985. doi: 10.1007/978-1-4899-2271-7

[53] Reeve J. A self-determination theory perspective on student engagement. Christenson SL, Reschly AL Wylie C. Handbook of research on student engagement. New York,NY: Springer. 2012; 149–172. doi: 10.1007/978-1-4614-2018-7_7

[54] Guay F, Vallerand RJ, Blanchard C. Motivation and Emotion. 2000;24(3):175–213. doi: 10.1023/a:1005614228250

[55] Van den Broeck A, Ferris DL, Chang C-H, Rosen CC. A Review of Self-Determination Theory’s Basic Psychological Needs at Work. Journal of Management. 2016;42(5):1195–229. doi: 10.1177/0149206316632058

[56] Niemann CC, Dickel P, Eckardt G. The interplay of corporate entrepreneurship, environmental orientation, and performance in clean‐tech firms—A double‐edged sword. Bus Strat Env. 2019;29(1):180–96. doi: 10.1002/bse.2357

[57] Baard PP, Deci EL, Ryan RM. Intrinsic Need Satisfaction: A Motivational Basis of Performance and Weil‐Being in Two Work Settings1. J Applied Social Pyschol. 2004;34(10):2045–68. doi: 10.1111/j.1559-1816.2004.tb02690.x

[58] Ryan RM, Deci EL. Self-Determination Theory: Basic Psychological Needs in Motivation, Development, and Wellness. Guilford Press. 2017. doi: 10.1521/978.14625/28806

[59] Ryan RM, Bernstein JH, Brown KW. Weekends, Work, and Well-Being: Psychological Need Satisfactions and Day of the Week Effects on Mood, Vitality, and Physical Symptoms. Journal of Social and Clinical Psychology. 2010;29(1):95–122. doi: 10.1521/jscp.2010.29.1.95

[60] Myers DG. The pursuit of happiness: Who is happy--and why. New York: W. Morrow. 1992. doi: 10.5860/choice.30-1790

[61] Eddie MWT, George DB, Hwee CE, Siew MD. Emotion and Appraisal Profiles of the Needs for Competence and Relatedness. Basic and Applied Social Psychology, 2009; 31:218–225. doi: 10.1080/01973530903058326

[62] Evelein F, Korthagen F, Brekelmans M. Fulfilment of the basic psychological needs of student teachers during their first teaching experiences. Teaching and Teacher Education. 2008;24(5):1137–48. doi: 10.1016/j.tate.2007.09.001

[63] Weiss H, Cropanzano R. Affective events theory. Res Organ Behav. 1996;18(1):1–74.

[64] Fredrickson BL. The role of positive emotions in positive psychology. The broaden-and-build theory of positive emotions. Am Psychol. 2001;56(3):218–26. doi: 10.1037//0003-066x.56.3.218 11315248 PMC3122271

[65] Isen AM. Positive affect and creativity. Bruner/Mazel. 1999.

[66] Deci EL, Ryan RM. Cognitive evaluation theory: perceived causality and perceived competence. 1985.

[67] Amabile TM, Schatzel EA, Moneta GB, Kramer SJ. Leader behaviors and the work environment for creativity: Perceived leader support. The Leadership Quarterly. 2004;15(1):5–32. doi: 10.1016/j.leaqua.2003.12.003

[68] Shalley CE, Perry-Smith JE. Effects of social-psychological factors on creative performance: the role of informational and controlling expected evaluation and modeling experience. Organ Behav Hum Decis Process. 2001;84(1):1–22. doi: 10.1006/obhd.2000.2918 11162295

[69] Shalley CE, Zhou J. Organizational Creativity Research. Handbook of Organizational Creativity. Psychology Press. 2024:3–32. doi: 10.4324/9781003573326-2

[70] Zhou J. Feedback valence, feedback style, task autonomy, and achievement orientation: Interactive effects on creative performance. Journal of Applied Psychology. 1998;83(2):261–76. doi: 10.1037/0021-9010.83.2.261

[71] Orakci Ş, Durnali M. The mediating effects of metacognition and creative thinking on the relationship between teachers’ autonomy support and teachers’ self‐efficacy. Psychology in the Schools. 2022;60(1):162–81. doi: 10.1002/pits.22770

[72] Gagné M, Deci EL. Self‐determination theory and work motivation. J Organ Behavior. 2005;26(4):331–62. doi: 10.1002/job.322

[73] Pajak EF, Glickman CD. Dimensions of School District Improvement. Educational Leadership. 1989;46(8):61–4.

[74] Deci EL, Ryan RM. Facilitating optimal motivation and psychological well-being across life’s domains. Canadian Psychology / Psychologie canadienne. 2008;49(1):14–23. doi: 10.1037/0708-5591.49.1.14

[75] Christina S, Latham GP. The Situational Interview as a Predictor of Academic and Team Performance: A Study of the Mediating Effects of Cognitive Ability and Emotional Intelligence. Int J Selection Assessment. 2004;12(4):312–20. doi: 10.1111/j.0965-075x.2004.00286.x

[76] Oldham GR, Cummings A. Employee creativity: personal and contextual factors at work. Academy of Management Journal. 1996;39(3):607–34. doi: 10.2307/256657

[77] Gong Y, Huang J-C, Farh J-L. Employee Learning Orientation, Transformational Leadership, and Employee Creativity: The Mediating Role of Employee Creative Self-Efficacy. AMJ. 2009;52(4):765–78. doi: 10.5465/amj.2009.43670890

[78] Frese M. Social support as a moderator of the relationship between work stressors and psychological dysfunctioning: a longitudinal study with objective measures. J Occup Health Psychol. 1999;4(3):179–92. doi: 10.1037//1076-8998.4.3.179 10431279

[79] Ford B, Kleiner BH. Managing engineers effectively. Bus. 1987;37(1):49–52.

[80] Deci EL, Eghrari H, Patrick BC, Leone DR. Facilitating internalization: the self-determination theory perspective. J Pers. 1994;62(1):119–42. doi: 10.1111/j.1467-6494.1994.tb00797.x 8169757

[81] Shalley CE, Zhou J, Oldham GR. The Effects of Personal and Contextual Characteristics on Creativity: Where Should We Go from Here?. Journal of Management. 2004;30(6):933–58. doi: 10.1016/j.jm.2004.06.007

[82] Liu D, Chen X-P, Yao X. From autonomy to creativity: a multilevel investigation of the mediating role of harmonious passion. J Appl Psychol. 2011;96(2):294–309. doi: 10.1037/a0021294 21058804

[83] Zhou J. When the presence of creative coworkers is related to creativity: role of supervisor close monitoring, developmental feedback, and creative personality. J Appl Psychol. 2003;88(3):413–22. doi: 10.1037/0021-9010.88.3.413 12814291

[84] Amabile TM, Barsade SG, Mueller JS, Staw BM. Affect and Creativity at Work. Administrative Science Quarterly. 2005;50(3):367–403. doi: 10.2189/asqu.2005.50.3.367

[85] George JM, Zhou J. Dual Tuning in a Supportive Context: Joint Contributions of Positive Mood, Negative Mood, and Supervisory Behaviors to Employee Creativity. AMJ. 2007;50(3):605–22. doi: 10.5465/amj.2007.25525934

[86] Deci EL, Connell JP, Ryan RM. Self-determination in a work organization. Journal of Applied Psychology. 1989;74(4):580–90. doi: 10.1037/0021-9010.74.4.580

[87] Breaugh JA. The Measurement of Work Autonomy. Human Relations. 1985;38(6):551–70. doi: 10.1177/001872678503800604

[88] Vallerand RJ, Pelletier LG, Blais MR, Briere NM, Senecal C, Vallieres EF. The Academic Motivation Scale: A Measure of Intrinsic, Extrinsic, and Amotivation in Education. Educational and Psychological Measurement. 1992;52(4):1003–17. doi: 10.1177/0013164492052004025

[89] Ryan RM, Deci EL. A self-determination theory approach to psychotherapy: The motivational basis for effective change. Canadian Psychology / Psychologie canadienne. 2008;49(3):186–93. doi: 10.1037/a0012753

[90] Sheldon KM, Gunz A. Psychological needs as basic motives, not just experiential requirements. J Pers. 2009;77(5):1467–92. doi: 10.1111/j.1467-6494.2009.00589.x 19678877

[91] Rauta V. Book Review: Culture Matters in Russia — and Everywhere: Backdrop for the Russia-Ukraine Conflict68.1489 HarrisonLawrence; YASINEvgeny, eds — Culture Matters in Russia — and Everywhere: Backdrop for the Russia-Ukraine Conflict (Lexington Book, 2015). Political Studies Review15(4), Nov. 2017: 635–636. International Political Science Abstracts. 2018;68(1):146–146. doi: 10.1177/002083451806800197

[92] Deci EL, Ryan RM, Williams GC. Need satisfaction and the self-regulation of learning. Learning and Individual Differences. 1996;8(3):165–83. doi: 10.1016/s1041-6080(96)90013-8

[93] Nie Y, Chua BL, Yeung AS, Ryan RM, Chan WY. The importance of autonomy support and the mediating role of work motivation for well-being: testing self-determination theory in a Chinese work organisation. Int J Psychol. 2015;50(4):245–55. doi: 10.1002/ijop.12110 25424389

[94] Chen B, Vansteenkiste M, Beyers W, Boone L, Deci EL, Van der Kaap-Deeder J, et al. Basic psychological need satisfaction, need frustration, and need strength across four cultures. Motiv Emot. 2014;39(2):216–36. doi: 10.1007/s11031-014-9450-1

[95] Watson D, Clark LA, Tellegen A. Development and validation of brief measures of positive and negative affect: the PANAS scales. J Pers Soc Psychol. 1988;54(6):1063–70. doi: 10.1037//0022-3514.54.6.1063 3397865

[96] Ye L, Chu X. Affect and creative performance: The mediating role of work engagement. soc behav pers. 2024;52(1):1–13. doi: 10.2224/sbp.12592

[97] George JM, Zhou J. Understanding when bad moods foster creativity and good ones don’t: the role of context and clarity of feelings. J Appl Psychol. 2002;87(4):687–97. doi: 10.1037/0021-9010.87.4.687 12184573

[98] Ye L, Sun H, Zhang J, Dong B, Chu X, Tao J, et al. Affect under need satisfaction and need thwarting: A new classification for the prediction of creative performance. Heliyon. 2024;10(10):e31323. doi: 10.1016/j.heliyon.2024.e31323 38813148 PMC11133818

[99] Baron RM, Kenny DA. The moderator-mediator variable distinction in social psychological research: conceptual, strategic, and statistical considerations. J Pers Soc Psychol. 1986;51(6):1173–82. doi: 10.1037//0022-3514.51.6.1173 3806354

[100] Hofstede G. Culture’s consequences: Comparing values, behaviors, institutions, and organizations across nations. International Educational and Professional. 2001.

[101] Liu Y, Raza J, Zhang J, Zhu N, Gul H. Linking autonomy support and health at work: The self-determination theory perspective. Curr Psychol. 2020;41(6):3651–63. doi: 10.1007/s12144-020-00884-0

[102] Yang S, Ok Choi S. Employee empowerment and team performance. Team Performance Management: An International Journal. 2009;15(5/6):289–301. doi: 10.1108/13527590910983549

[103] Xu Y, Li Y, Nugroho H, Delaney JT, Luo P. Meta-analysis of the Factors Influencing the Employees’ Creative Performance. In Proceedings of the Eleventh International Conference on Management Science and Engineering Management 11. 2018;658–669. Springer International Publishing. doi: 10.1007/978-3-319-59280-0_54

[104] Vallerand RJ, Pelletier LG, Blais MR, Briere NM, Senecal C, Vallieres EF. On the Assessment of Intrinsic, Extrinsic, and Amotivation in Education: Evidence on the Concurrent and Construct Validity of the Academic Motivation Scale. Educational and Psychological Measurement. 1993;53(1):159–72. doi: 10.1177/0013164493053001018

[105] Nili F, Tasavori M. Linking an autonomy-supportive climate and employee creativity: the influence of intrinsic motivation and company support for creativity. EBR. 2022;34(5):666–88. doi: 10.1108/ebr-06-2021-0146

[106] Ni Y-X, Wu D, Bao Y, Li J-P, You G-Y. The mediating role of psychological needs on the relationship between perceived organizational support and work engagement. Int Nurs Rev. 2023;70(2):204–10. doi: 10.1111/inr.12797 35962469

[107] Cohen S, Wills TA. Stress, social support, and the buffering hypothesis. Psychological Bulletin. 1985;98(2):310–57. doi: 10.1037/0033-2909.98.2.310 3901065

[108] Fischer A. Gender and emotion: social psychological perspectives. Cambridge University Press. 2000.

[109] Naghavi F, Redzuan M. The relationship between gender and emotional intelligence. World Applied Sciences Journal, 2011;15(4):555–561. doi: 10.15373/2249555x/sept2013/169

[110] Gagné M, Forest J, Vansteenkiste M, Crevier-Braud L, van den Broeck A, Aspeli AK, et al. The Multidimensional Work Motivation Scale: Validation evidence in seven languages and nine countries. European Journal of Work and Organizational Psychology. 2014;24(2):178–96. doi: 10.1080/1359432x.2013.877892

[111] Deci EL, Olafsen AH, Ryan RM. Self-Determination Theory in Work Organizations: The State of a Science. Annu Rev Organ Psychol Organ Behav. 2017;4(1):19–43. doi: 10.1146/annurev-orgpsych-032516-113108

